# Effect of Suckling Management and Ewe Concentrate Level on Methane-Related Carbon Footprint of Lamb Meat in Sardinian Dairy Sheep Farming

**DOI:** 10.3390/ani11123605

**Published:** 2021-12-20

**Authors:** Gianni Battacone, Mondina Francesca Lunesu, Salvatore Pier Giacomo Rassu, Anna Nudda, Giuseppe Pulina

**Affiliations:** Dipartimento di Agraria, Sezione di Scienze Zootecniche, University of Sassari, Viale Italia 39, 07100 Sassari, Italy; battacon@uniss.it (G.B.); pgrassu@uniss.it (S.P.G.R.); anudda@uniss.it (A.N.); gpulina@uniss.it (G.P.)

**Keywords:** carbon footprint, suckling lamb, extensive system, Mediterranean region

## Abstract

**Simple Summary:**

Suckling lamb meat is the secondary product of the Mediterranean traditional dairy sheep industry. Similar to the main production, i.e., milk, lamb meat contributes to the emission of greenhouse gases (GHG), whose main portion is represented by enteric methane produced by the lamb dams. Such an emission, although limited in quantitative terms, should be mitigated by appropriate feeding or compensation techniques. Among all the sources of variation of meat lamb emissions, sex of the lamb and type of lambing (single or twins) showed the largest effect.

**Abstract:**

The aim of this study was to estimate the methane-linked carbon footprint (CF) of the suckling lamb meat of Mediterranean dairy sheep. Ninety-six Sarda dairy ewes, divided into four groups of 24 animals each, were assigned to 2 × 2 factorial design. The experiment included the suckling lamb feeding system: traditional (TS), in which lambs followed their mothers on pasture during grazing time, vs. separated (SS), in which lambs remained indoors, separated from their mothers during the grazing time. Each group was divided into high (HS) and low (LS) supplemented ewes (600 g/d vs. 200 g/d of concentrate). The estimated CH_4_ emission of the ewes, calculated per kg of body weight (BW) gain of the lamb during the suckling period, was then converted to CO_2_eq with multiplying factor of 25. The TS lambs showed lower methane-linked emissions than SS ones (*p* < 0.05). The sex of lambs affected their methane-linked CF, with males having lower (*p* < 0.05) values than females. Twins displayed much lower methane-linked CF than singles (4.56 vs. 7.30 kg of CO_2_eq per kg of BW gained), whereas the level of supplementation did not affect greenhouse gases (GHG) emission. Interaction displayed lower and not-different GHG emissions for both indoor- and outdoor-reared twins. In conclusion, the methane-linked CF of the suckling lamb meat can be reduced by maintaining the traditional lamb rearing system and by improving flock prolificacy.

## 1. Introduction

Dairy lamb is a secondary product of dairy sheep farms, and it is consumed mostly in Mediterranean countries [1]. It represents a niche product appreciated by consumers for its nutritional and organoleptic characteristics, due to both the young slaughtering age (4–6 weeks of age) and the quality of maternal milk obtained mainly by grazing natural pastures [2]. Moreover, the suckling lamb meat is an interesting source of fatty acids of nutritional importance [3] and it is particularly suitable for children’s diets, especially in the weaning phase [4,5].

Growing concerns of European citizens about the environmental impact of animal productions require that foods must also guarantee sustainability, especially in terms of climate-altering gas emissions [6]. As a consequence, the number of livestock life cycle assessment (LCA) studies has considerably increased in the last two decades [7]. Most of this research deals with the environmental sustainability of beef and pork production. Carbon footprint (CF) of lamb meat has received less attention [7], and studies were carried out mostly on the quantification of environmental performance of the heavy lamb with values ranging hugely from 2.8 [8] to 38.45 [9] kg CO_2_eq per kg of live weight (LW) [8,9,10,11,12,13,14,15,16,17,18]. All research was carried out in Oceania (most of the studies), in Europe, US, China, and Chile [19]. Most of them quantified greenhouse gases (GHG) emissions by comparing different farming systems, from pasture to zero-grazing [13], from lowland to hill farms [14], from conventional to organic [10], or considering different forage species in pasture-based flock management [20]. Recently, methane production of fattening lambs was predicted by intramuscular fatty acid profile [21].

Most of these studies consider 1 kg of live weight (LW) as functional unit and, since sheep farms produce two or three coproducts (milk, meat, and wool), economic or a biophysical allocation are generally used to distribute the overall impacts between them [19]. Furthermore, the greater amount of impact in this meat production system occurs at the farm level (90% [9,22]), and it depends mostly on enteric methane emissions whose relative contribution to total CF ranges from 58% [13] to 80%% [23]. CO_2eq_ emissions from purchased feeds, energy, and fuel, and N_2_O emissions from soil and manure management, contribute to the total impact in a lesser proportion. 

All of these studies deal with meat sheep lamb production, whereas, to the best of the authors’ knowledge, the CF of suckling dairy lambs has not been estimated. As recently reviewed by Battacone et al. [3], suckling lambs are fed exclusively maternal milk from birth to slaughter. Thus, this type of production does not require additional inputs than those demanded by their mothers. For these reasons, CF of suckling dairy lamb should be probably lower than values available in the literature.

Feeding technique has been demonstrated to be effective in reducing the CF of milk in dairy cows [24,25,26], goats [25,27], and sheep [25,26,27]. Methane emission per unit of milk or meat has continuously decreased during the last decades, and it is expected to continue this trend [28]. Thus, it is reasonable to hypothesize that feeding techniques implemented to reduce the environmental impact of dairy ewes could also influence the CF of suckling lamb meat. In fact, the feeding regimen influences ewe dry matter intake (DMI) and, consequently, the methane yield (the principal GHG produced by sheep). 

In this work the CF of suckling lambs under different management systems and mother feeding was estimated by considering exclusively the CO_2_eq derived from the CH_4_ emitted by the ewes during the suckling period. This choice assumed that methane emissions related to gestation and replacement can be totally attributed to milk production, which is the main activity of the farm, with an allocation of 100% of all other emissions to this production. Such a strong assumption was made in order to simplify calculations and to make the comparison between different production systems easier, without altering the overall impacts of sheep dairy farms. 

Aim: The aim of this work was to estimate the methane-linked carbon footprint (CF) of the suckling lamb meat of Mediterranean dairy sheep.

## 2. Materials and Methods

The CF of suckling lambs was estimated using data collected in a commercial dairy sheep farm located in the northwest of Sardinia (Italy). The animal protocol was carried out in compliance with the EU and Italian regulation on animal welfare. For this study, no animals were specifically killed for experimental purposes; however, data at commercial slaughter were collected. University ethics approval was also not required. 

### 2.1. Experimental Procedure: Animals and Diet

The experiment involved ninety-six Sarda nursing ewes (body weight (BW): 46.33 ± 0.40 kg; mean ± standard error) who were monitored with their lambs for a period of 28 days. The trial started immediately after lambing. In Sardinian dairy sheep farming, there are two lambing seasons: autumn for pluriparous, and spring for primiparous. In this trial, only pluriparous ewes were chosen, so lambing was concentrated at mid-November.

A sample of 100 ewes lambing with parturition occurring within two days were selected from the flock and serially numbered. Then, 96 animals were extracted and randomly assigned to four groups of 24 animals each in a 2 × 2 factorial design:

(a) Traditional system with high supplementation (TS-HS), in which mothers were followed by suckling lambs during the grazing time and they received a high dose of supplement (600 g/d of concentrate).
(b) Traditional system with low supplementation (TS-LS), in which mothers were followed by suckling lambs during the grazing time and they received a low dose of supplement (200 g/d of concentrate).
(c) Separated system with high supplementation (SS-HS), in which mothers were not followed by suckling lambs during the grazing time (suckling lambs remained indoors) and they received a high dose of supplement (600 g/d of concentrate).
(d) Separated system with low supplementation (SS-LS), in which mothers were not followed by suckling lambs during the grazing time (suckling lambs remained indoors) and they received a low dose of supplement (200 g/d of concentrate).

The ewes grazed daily on a lush pasture for 6 h (9:30 a.m. to 15:30 p.m.). The concentrate was offered during two daily meals. In addition, all ewes had ad libitum access to hay during the night. The chemical composition of feeds offered is showed in Table 1.

The newborn lambs (*n* = 44 females and 52 males) were fed exclusively maternal milk throughout the whole experimental period (28 days). At 28 days of age, they were weighed and then slaughtered in an authorized commercial abattoir.

### 2.2. Measurements and Sampling

During the experimental period, BW of ewes was measured weekly by using an electronic scale. Lamb weight was measured at birth and then once a week until slaughter. Average daily gain was calculated.

Individual milk yield was measured on the two consecutive days after slaughter (two times per day, at morning and evening milking) to confirm the estimation of milk produced by the dams in function of daily growth of lambs. Individual milk samples (*n* = 384; 96 per treatment) were also collected and analyzed for chemical composition. 

Samples of grass, hay, and concentrate were collected weekly for chemical analysis.

### 2.3. Chemical Analyses

Milk samples were analyzed for fat, protein, lactose (infrared method; Milkoscan 4000, Foss Eletric, Hillerød, Denmark), urea content (enzymatic-colorimetric method based on Berthelot reaction; Chemspec 150, Bentley Instruments Inc., Chaska, MN, USA), and somatic cell count (SCC, flow-cytometry method; Fossomatic 5000, Foss Electric, Hillerød, Denmark).

Feed samples were ground with a Hammer mill by using a 1 mm screen, and then analyzed for DM, CP (Kjeldahl method; AOAC International, [29]; method 988.05), NDF, ADF, ADL (including termostable-amylase and following the method of Van Soest et al. [30]), ether extract (EE; Soxlet, AOAC International, [31]; method 920.39), and ash (AOAC International, [29]; method 942.05) after drying at 105 °C.

### 2.4. Carbon Footprint Assessment, System Boundary, Functional Unit, and Allocation Method

The CH_4_-linked carbon footprint was calculated within a cradle to farm gate system boundary considering maternal enteric CH_4_ emissions and milk suckled by the lambs as the main emissions hotspots. Data collected during the experimental trial were used to estimate maternal DMI [32] as follows:

I = −0.545 + 0.095 MW + 0.65 FPCM + 0.0025 BWC(1)

where I = DMI in kg/head day^−1^; MW = metabolic weight (BW^0.75^) in kg; BWC = bodyweight change in g/day; FPCM = fat (F = 6.5%) and protein (*p* = 5.8%) corrected milk (M) in kg which, in turn, was calculated as:

FPCM = M (0.25 + 0.085F + 0.035P) (kcal/kg = 1047) (2)

where M = milk yield in kg; F and *p* = fat and protein concentration in %, [33].

Milk suckled by the lamb was estimated at 5.376 kg/kg of BW growth (BWG), arranging the Pulina et al. [34] equation which estimates the daily milk production of dams (M) in g/day as function of BWG (in g/day) and MBW of lambs (in kg):

M = 140.6 + 4.52 BWG − 0.705 MBW (3)

Methane emissions were then estimated by using the equation 10.21 of the Intergovernmental Panel on Climate Change (IPCC) guidelines for national GHG inventories [35]:

EF = GE(Ym/100)/55.65(4)

where EF = emission factor, kg CH_4_ head^−1^; GE = gross energy intake, MJ head^−1^ day^−1^; Ym = methane conversion factor, per cent of gross energy in feed converted to methane; the factor 55.65 (MJ/kg CH_4_) is the energy content of methane. 

The estimated CH_4_ emission of the ewes was then expressed in terms of CO_2_eq where 1 kg CH_4_ = 25 kg CO_2_eq in accordance with the global warming potential of emissions defined by the IPCC guidelines. Finally, the CH_4_-linked CF was calculated considering 1 kg of BW gain (during the suckling period) as functional unit (FU) and applying no allocation factor.

### 2.5. Statistical Analysis

Ewe DMI data were analyzed with the following linear model:

DMI = µ + G + C + P + 1st_inter + ε

where DMI (in kg) is the total 28 days intake during the suckling period, G is the lamb management (TS vs. SS), C is the supplement level (HS vs. LS), P is the kind of lambing (single vs. twins, no triplets were admitted to the experiment), and 1st_inter are the first order interactions between the couples of experimental factors.

Birth weight, slaughter weight, average daily gain (ADG), and CH_4_-linked CF of lambs were analyzed with the following linear model:

Y = µ + G + C + P + S + 1st_inter + ε

where Y is the dependent variable, G is lamb management (TS vs. SS), C is the supplement level (HS vs. LS), P is the kind of lambing (single vs. twins, no triplets were admitted to the experiment), S is the sex of lamb, and 1st_inter are the first order interactions between the couples of experimental factors.

Differences between means were detected with Tukey test, and significative level was declared for *p* < 0.05 [36].

## 3. Results and Discussion

Milk production of ewes in the first control after lamb slaughter were 1.36 ± 0.074 kg/d (mean ± standard error) for TS-HS group, 1.19 ± 0.075 kg/d for TS-LS group, 1.28 ± 0.074 kg/d for SS-HS group, and 1.32 ± 0.075 kg/d for SS-LS group. They were slightly lower than those estimated by using Equation (3) (TS-HS: 1.68 ± 0.095 kg/d, TS-LS: 1.46 ± 0.069 kg/d, SS-HS: 1.32 ± 0.092 kg/d, SS-LS: 1.35 ± 0.068 kg/d; mean ± standard error) because milking normally produces slightly less milk than suckling. 

### 3.1. Suckling Lambs Performance at Birth and after the Suckling Period

Data on birth body weight, body weight at slaughter, and ADG are shown in Table 2. 

The first-order interactions between the couples of experimental factors were never significant (*p* > 0.05), except for lamb management x type of lambing interaction for kg CO_2_eq/kg lamb BW gain. Mother’s supplement level did not affect lamb birth weight. The lamb management significantly affected body weight at slaughter (*p* < 0.01) and the ADG (*p* < 0.001). TS Lambs exhibited higher slaughter weights and ADG compared to SS lambs, probably because they could suckle more times a day from their mother than SS. Such a larger amount of milk received by TS lambs compensated the higher energy expenditure for movement and thermoregulation needed to follow the mothers during grazing.

Concerning the type of lambing, our data evidenced that single-born lambs had higher birth weight (*p* < 0.01), birth weight at slaughter (*p* < 0.001), and ADG (*p* < 0.01) compared to twins. These results are comparable to those observed in previous studies in which single lambs showed higher live weight than twin lambs both at birth and later on [37,38], and tended to grow faster than those with lower live weight at birth [38]. 

Regarding the sex, male lambs had higher birth weight (*p* < 0.01), birth weight at slaughter (*p* < 0.01), and ADG (*p* < 0.01) compared to female lambs. Generally, at birth, males show higher birth weight than females [39,40,41]; this difference has been reported for several breeds, and it seems to persist during the life [42]. Other studies did not find differences between sexes at birth, but they evidenced that male lambs tend to grow faster than females [38]. 

### 3.2. Carbon Footprint of Suckling Lambs

**Figure 1:**
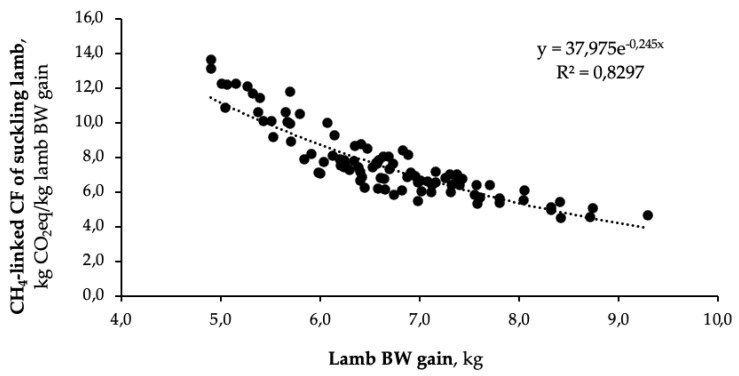
Allometric relation between CH_4_-linked carbon footprint (CF) of suckling lamb and lamb body weight (BW) gain.

In the present study, CH_4_-linked CF of suckling lamb varied between 4.56 and 7.30 kg CO_2_eq/kg lamb BW gain, respectively. A comparison with other studies is quite difficult because of the lack of research on the estimation of CF of suckling lambs and also because of the methodological heterogeneity among studies. However, from our data, it is possible to observe a strong relationship between CH_4_-linked CF of suckling lamb and BW gain of lambs (Figure 1), in agreement with the relationships between GHG emissions and the kg of LW or BW gain observed in sheep meat breeds [43,44,45,46].

The estimation of total DMI of ewes and CH_4_-linked CF of suckling lambs are reported in Table 3. 

**Figure 2:**
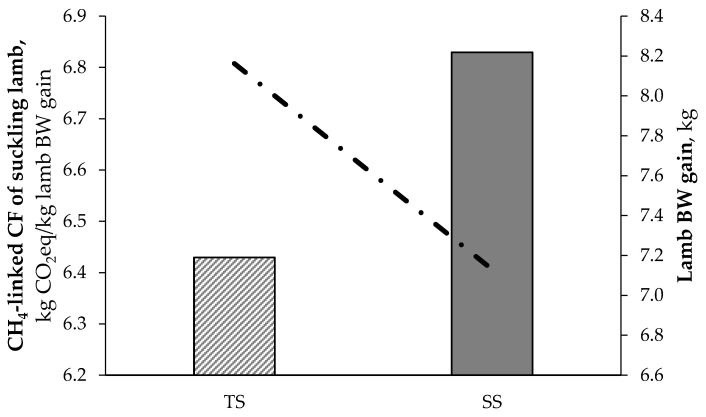
Methane-linked carbon footprint (CF) and lamb body weight (BW) gain in suckling lambs raised in the traditional (TS) and separated systems (SS).

Ewes of TS showed higher (*p* < 0.01) total DMI as a consequence of their higher milk production, caused by the higher frequency of suckling activities of the lambs. In fact, lambs following their mothers all day around matched their ethological behavior, compared to lambs kept indoors that had the possibility to suckle only during the night. Increasing the frequency of suckling in TS lambs caused more frequent udder-emptying, which stimulates milk secretion [47]. The lower CH_4_-linked CF of TS lambs compared to SS (*p* < 0.05) could be therefore explained by the fact that the higher DMI of mothers is diluted in a greater BW gain of their offspring (Figure 2).

The level of supplement used did not affect the DMI of ewes or the CH_4_-linked CF of suckling lambs. In this sense, very few studies have evaluated the effect of concentrate supplementation in animals under grazing conditions. Commonly, a negative relationship between CH_4_ (g/kg DMI) and level of concentrate in the diet has been reported in beef cattle [48] and lambs [49], and the use of concentrates has been proposed as a valid mitigation strategy for ruminants [50]. In the current study, the results are likely explained by the lack of substitution effect of supplement on pasture, because of the similar nutrient composition of grass and supplement. This is in agreement with a study conducted in dairy cows under high-quality grazing conditions where the increase in concentrate supplementation resulted in a simultaneous increase in enteric methane emissions and milk production, and so considering that methane emissions are expressed per unit of milk yield, the effect of supplement on GHG mitigation was not evident [51].

Previous studies carried out in lambs evidenced that varying the proportion of concentrate did not affect CH_4_ emissions in lambs fed a basal diet composed of high-quality forages [52]. This suggests that the positive reduction of CH_4_ per kg of DMI due to an increase of concentrate amount in the diets could be, in part, counterbalanced by the higher intake of high-quality forages. The findings of the present study suggest that high-quality pasture could act in the same way as concentrates in reducing CH_4_ emissions. In fact, several studies conducted on beef cattle [53] and sheep [54] farmed in grazing systems with different pasture quality evidenced that high-quality grass can reduce CH_4_ emissions per unit of DMI (CH_4_/kg DMI) in comparison with low-quality grass. The positive effect of high-quality pasture on the reduction of enteric CH_4_ emissions is due to the lower content of NDF, to the high content of CP, and to the higher digestibility [55]. The quantity of fresh forages offered can also have an effect: in fact, CF is lower in high, rather than in low, productive grazing systems [9]. Thus, the improvement of pasture (both in quality and quantity) can be considered a good mitigation strategy to implement at a farm level for reducing enteric methane emissions of ruminants under grazing condition [50,55]. In addition, the use of pasture contributes to reducing the consumption of off-farm feeds and the management of pasture increases soil’s carbon sequestration [23].

**Figure 3:**
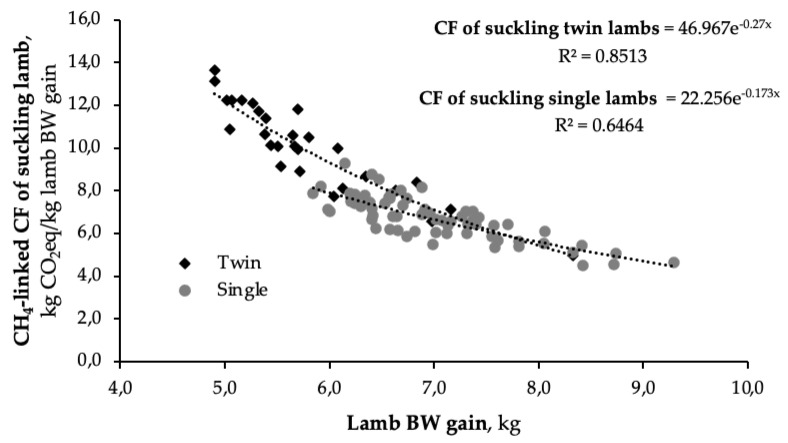
Allometric relation between CH4-linked carbon footprint (CF) of suckling lamb and body weight (BW) gain of twins and single-born lambs.

Twin-lambing ewes showed higher (*p* < 0.001) DMI than ewes with single lambs, probably because of their higher milk production. It is widely assessed that ewes with twins produce more milk than ewes with single lambs [56]. This is due to both the action of the placental lactogenic hormone, whose secretion is proportional to the weight of the placenta and that stimulates greater mammary growth [57], and to the more frequent and complete emptying of the mammary gland [58,59]. As twin-lambing ewes produced more milk, the CH_4_-linked CF of their lambs was markedly lower than that of single lambs; this result is also due to sharing of the maintenance requirements of mothers between the twins. The relation between CH4-linked CF of suckling lambs and lamb BW gain in twins compared to single-born lambs is shown in Figure 3.

**Figure 4:**
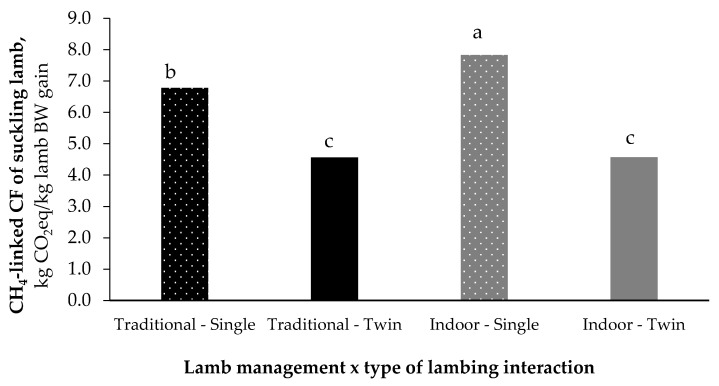
Effect of lamb management x type of lambing interaction on CH_4_-linked CF (expressed on kg CO_2_eq/kg lamb BW gain). ^a,b,c^ mean values with different superscripts differ (*p* < 0.05) for the interaction between lamb management and type of lambing interaction.

The CH_4_-linked CF of twins did not change with the management system, while that of single lambs was lower when they followed the mother on pasture, as evidenced by the significant interaction (*p* < 0.05; Figure 4).

The sex of lambs affects their CH_4_-linked CF, with males having lower (*p* = 0.038) values than those of females, due to their highest growth rates which dilute the CH_4_ emission of the mother into a higher BW at slaughter, as previously shown in Figure 1.

### 3.3. Practical Implications

Since Sarda sheep are fed mainly on pasture, our data suggest that the environmental impact of suckling lamb meat production can be reduced by improving flock prolificacy and maintaining the traditional lamb rearing system. However, these results are affected by the high nutritional value of pasture and of its large availability. 

The improvement of forage quality (through the evaluation of the best phenological stage) and forage type (in terms of botanical composition) can be considered as valid mitigation strategies to reduce livestock emissions [26]. Pasture-based systems in the dairy sheep industry are an important tool to mitigate the GHG impact for the capacity of grasslands to sequester C in soil as well as for the ability to provide ecosystem service and animal welfare [26,60,61,62].

Considering the high diffusion of agro–silvo–pastoral systems in Mediterranean countries where dairy sheep are farmed, new findings on carbon sequestration in soil under pasture management demonstrate that the GHG emissions for suckling lamb meat production can be compensated annually by few m^2^ of undisturbed pastureland [63].

## 4. Conclusions

Growing concerns about GHG emissions among consumers are driving supply chains to reduce their impacts until the net zero goal is achieved. Not even niche productions, such as dairy lambs linked to traditional pastoral systems, widespread in the Mediterranean area escape this logic. This work, which evaluated only methane emissions from lactating ewes as representative of the GHG impact of dairy lamb meat production, evidenced that the type of suckling management, but not the ewe concentrate level, affected the CH_4_-linked CF of lamb meat. Specifically, traditional suckling techniques resulted in a lower CH_4_-linked CF of lambs compared to one in which the lambs were separated from their mother during the grazing period. Moreover, a high twinning rate of the flock can be an effective option for reducing the GHG impact.

To conclude, this paper provides the first data on the estimation of environmental impact of the suckling lamb meat production in the Mediterranean region and suggests that to reduce the environmental impact of suckling lamb production systems, lambs could be raised with traditional suckling technique and should be twins.

Some agronomic and livestock practices can be linked to mitigate the GHG impact of dairy sheep industry.

## Figures and Tables

**Table 1:** Chemical composition of feeds offered to nursing ewes.

	Concentrate ^1^	Hay	Pasture
DM, % as fed	87.2	91.0	22.3
CP, % on DM	17.0	5.0	18.2
NDF, % on DM	41.5	61.0	42.5
ADF, % on DM	20.7	50.0	25.2
ADL, % on DM	5.3	5.0	3.0
Ash, % on DM	9.8	7.9	10.4
EE, % on DM	2.4	1.9	1.5

DM = dry matter; CP = crude protein; NDF = neutral detergent fiber; ADF = acid detergent fiber; ADL = acid detergent lignin; EE = ether extract. ^1^ The concentrate was composed of the following ingredients: wheat bran, soybean hulls (from genetically modified soybean), alfalfa meal, wheat distilled dried grains, wheat bran, sunflower extraction meal, maize germ cake, dried sugar beet pulp, hydrogenated vegetable fatty acid, corn gluten meal, molasses sugar beet, calcium carbonate from limestone rocks, soybean cake, maize. Vitamin supplement: A, 15,000 U/kg; D3 2923, U/kg; E, 30 mg/kg; B12, 0.06 mg/kg. Minerals supplement: Fe (FeSO_4_), 35 mg/kg; iodine (Ca(IO_3_)_2_), 1.1 mg/kg; MnO, 70 mg/kg; Se (Na_2_SeO_3_), 0.51 mg/kg; ZnO, 70 mg/kg; Mo (Na_2_MoO_4_), 1.0 mg/kg.

**Table 2:** Least-squares means for initial body weight (birth weight), final body weight (at slaughter after 28 days suckling period), and average daily gain (ADG) of Sarda suckling lambs.

	Birth Weight (kg)		Slaughter Weight (kg)		ADG (kg/d)	
	Mean	SE ^1^	Mean	SE	Mean	SE
Lamb Management						
Traditional	3.98	0.08	10.20	0.20	0.22	0.006
Indoor	4.03	0.08	9.37	0.21	0.19	0.007
*p*-value	0.575		0.004		<0.001	
Ewes’ Supplement Level						
Low (200 g/d/ewe)	3.90	0.08	9.78	0.21	0.21	0.007
High (600 g/d/ewe)	4.10	0.08	9.79	0.21	0.20	0.007
*p*-value	0.812		0.170		0.158	
Type of Lambing						
Single	4.28	0.06	10.82	0.17	0.23	0.005
Twins	3.73	0.04	8.76	0.27	0.18	0.008
*p*-value	<0.001		<0.001		<0.001	
Sex of Lamb						
Male	4.12	0.080	10.17	0.20	0.22	0.006
Female	3.88	0.084	9.40	0.22	0.20	0.007
*p*-value	0.010		0.038		0.225	
Mean ^2^	4.14	0.061	10.32	0.18	0.22	0.005

^1^ SE = standard error; ^2^ Arithmetic mean.

**Table 3:** Least-squares mean for total dry matter intake (DMI) of ewes and CH_4_-linked carbon footprint of lambs (expressed in kg of CO_2_eq/kg of BW gain) during 28-day suckling period.

	Total DMI of Ewes (kg)		kg CO_2_eq/kg Lamb BW Gain	
	Mean	SE ^1^	Mean	SE
Lamb Management				
Traditional	63.26	1.03	5.67	0.15
Indoor	58.42	1.02	6.20	0.16
*p*-value	0.001		0.016	
Ewes’ Supplement Level				
Low (200 g/d/ewe)	60.83	1.13	5.77	0.16
High (600 g/d/ewe)	60.85	0.95	6.10	0.15
*p*-value	0.988		0.137	
Type of Lambing				
Single	50.79	0.78	7.30	0.15
Twins	66.89	1.25	4.56	0.17
*p*-value	<0.001		<0.001	
Sex of Lamb				
Male			5.70	0.16
Female			6.16	0.16
*p*-value			0.038	

^1^ SE = standard error.

## Data Availability

The data presented in this study are available on request from the corresponding author.
